# Three-Dimensional Normal Human Neural Progenitor Tissue-Like Assemblies: A Model of Persistent Varicella-Zoster Virus Infection

**DOI:** 10.1371/journal.ppat.1003512

**Published:** 2013-08-01

**Authors:** Thomas J. Goodwin, Maureen McCarthy, Nikolaus Osterrieder, Randall J. Cohrs, Benedikt B. Kaufer

**Affiliations:** 1 Disease Modeling/Tissue Analogues Laboratory, NASA Johnson Space Center, Houston, Texas, United States of America; 2 Institut für Virologie, Freie Universität Berlin, Berlin, Germany; 3 Department of Neurology, University of Colorado School of Medicine, Aurora, Colorado, United States of America; University of Glasgow, United Kingdom

## Abstract

Varicella-zoster virus (VZV) is a neurotropic human alphaherpesvirus that causes varicella upon primary infection, establishes latency in multiple ganglionic neurons, and can reactivate to cause zoster. Live attenuated VZV vaccines are available; however, they can also establish latent infections and reactivate. Studies of VZV latency have been limited to the analyses of human ganglia removed at autopsy, as the virus is strictly a human pathogen. Recently, terminally differentiated human neurons have received much attention as a means to study the interaction between VZV and human neurons; however, the short life-span of these cells in culture has limited their application. Herein, we describe the construction of a model of normal human neural progenitor cells (NHNP) in tissue-like assemblies (TLAs), which can be successfully maintained for at least 180 days in three-dimensional (3D) culture, and exhibit an expression profile similar to that of human trigeminal ganglia. Infection of NHNP TLAs with cell-free VZV resulted in a persistent infection that was maintained for three months, during which the virus genome remained stable. Immediate-early, early and late VZV genes were transcribed, and low-levels of infectious VZV were recurrently detected in the culture supernatant. Our data suggest that NHNP TLAs are an effective system to investigate long-term interactions of VZV with complex assemblies of human neuronal cells.

## Author Summary

Varicella-zoster virus (VZV), the alphaherpesvirus that typically causes childhood chickenpox and shingles in adults, becomes latent in neurons, thus remaining in the body for a lifetime. Unfortunately, few models are available to study the establishment of VZV latency since the virus infects only humans and establishes persistent infections and latency only in neurons, a slowly proliferating, short-lived cell in culture. We have successfully maintained normal human neural progenitor cells (NHNP) in tissue-like assemblies (TLAs) in 3-dimensional (3D) cultures for up to 6 months. The 3D NHNP TLAs show some characteristics as those found in the human trigeminal ganglia, the site of VZV latency. NHNP TLAs infected with VZV remain viable for 3 months during which time VZV DNA replicates and remains genetically stable, virus genes are transcribed, and infectious VZV is sporadically released. The ability to maintain VZV infected NHNP cells in culture for extended times provides the unique opportunity to study the molecular interactions between this important human pathogen and neuronal tissue to an extent previously unattainable.

## Introduction

Varicella-zoster virus (VZV) is a ubiquitous human herpesvirus, as evidenced by a seroprevalence of more than 95% worldwide [1]. Among human herpesviruses, VZV has the smallest genome of approximately 125 kbp, which contains at least 70 open reading frames (ORFs) and consists of two unique regions, unique long (U_L_) and unique short (U_S_), each flanked by inverted repeat regions (TR_L_, IR_L_, TR_S_, IR_S_) [2], [3]. Primary VZV infection typically causes childhood varicella (chickenpox). During primary infection the virus gains access to and establishes latency in multiple cranial, dorsal root and autonomic ganglia. Varicella vaccination programs have successfully reduced the incidence of clinical disease in children in the USA by about 80% [4], [5]; however, the attenuated vaccine, like wild-type VZV, is still able to establish latency in the peripheral nervous system [6]. Both wild-type and vaccine virus can reactivate from latency, particularly in elderly and immunocompromised individuals. Reactivation is associated with a declining VZV specific T-cell immunity and can result in herpes zoster (shingles) especially in the elderly, which is characterized by severe pain and often followed by postherpetic neuralgia [7], [8]. Reactivation can also lead to progressive outer retinal necrosis and stroke by ischemic vasculopathy [9], [10]. With respect to disease severity, duration and quality-of-life impairment, VZV reactivation in adulthood can be more serious than primary childhood infection.

VZV pathogenesis, latency, and reactivation are difficult to study, as the virus exclusively infects humans and no animal model is currently available to investigate VZV latency. As a result, our knowledge of VZV latency is based on the analysis of human ganglia removed at autopsy. However, primary human ganglia have several disadvantages: (i) they can only be cultured for a few days *in vitro*, (ii) the availability of human ganglia is limited, (iii) the time between death and tissue collection cannot be predetermined, (iv) there are considerable human to human variations including preexisting virus burden and time since primary VZV infection, and (v) since most ganglia explants contain latent VZV, it is difficult to perform prospective studies using VZV with defined genetic mutations. Therefore, our understanding of VZV latency largely represents a snapshot of human ganglia that show a high degree of variability [11] taken shortly after death.

Human fetal dorsal root ganglia (DRG) are typically free of latent VZV. Enzymatically dissociated human DRG show neurite outgrowth when maintained in culture and have facilitated the analysis of apoptosis inhibition mediated by VZV in neurons [12], [13]; however, since the cultures can be maintained for only a few weeks, their application to study latent infection is limited. Human fetal DRG can be maintained for eight weeks as xenografts under the kidney capsule of SCID mice [14], and the virus gene expression profile at eight weeks post-infection in DRG xenografts is comparable to latently infected adult trigeminal ganglia; however, difficulties in maintaining viable DGR xenografts along with ethical concerns regarding fetus-derived tissue has limited their widespread utilization.

Human stem cells provide an alternative source of neurons. Stem cell-derived neurospheres can be maintained in tissue culture and in lateral ventricles of neonatal non-obese diabetic SCID mice. While human neurospheres maintained in mice can be infected with VZV and show no obvious virus-induced cytopathic effect, time course experiments using this system are technically challenging and costly [15]. In addition, differentiated human pluripotent stem cells can be infected with VZV and are useful to visualize retrograde virus axonal transport; however in these cultures, VZV initiates lytic replication that appears to be related to the neuronal purity [16], [17], [18]. Neuronal cultures lacking significant numbers of glia or astrocytes can be infected, show no virus-induced cytopathic effects (CPE) and do not produce infectious virus; but, it is challenging to obtain these short-lived cultures of pure neurons [19]. Taken together, the available neuronal models to study VZV interactions are hindered by their limited availability, elaborate maintenance requirements, limited lifespan, neuronal purity and significant ethical concerns.

In order to overcome these problems, we developed a novel three-dimensional (3D) culture system to maintain partially differentiated normal human neural progenitor (NHNP) cells as tissue-like assemblies (TLAs) that share some features with neurons found in human trigeminal ganglia. The 3D NHNP TLAs can be maintained for at least three months following VZV infection. During this time, VZV DNA was readily detected, VZV immediate-early, early and late genes were transcribed, no VZV-induced CPE was observed, but low amounts of infectious virus were released into the culture medium. Importantly, the VZV genome was stable throughout the three month infection period, suggesting that the 3D NHNP TLA culture system may be useful to investigate the long-term interplay between VZV and neural tissue.

## Results

### NHNP TLAs 3D cell cultures

**Figure 1:**
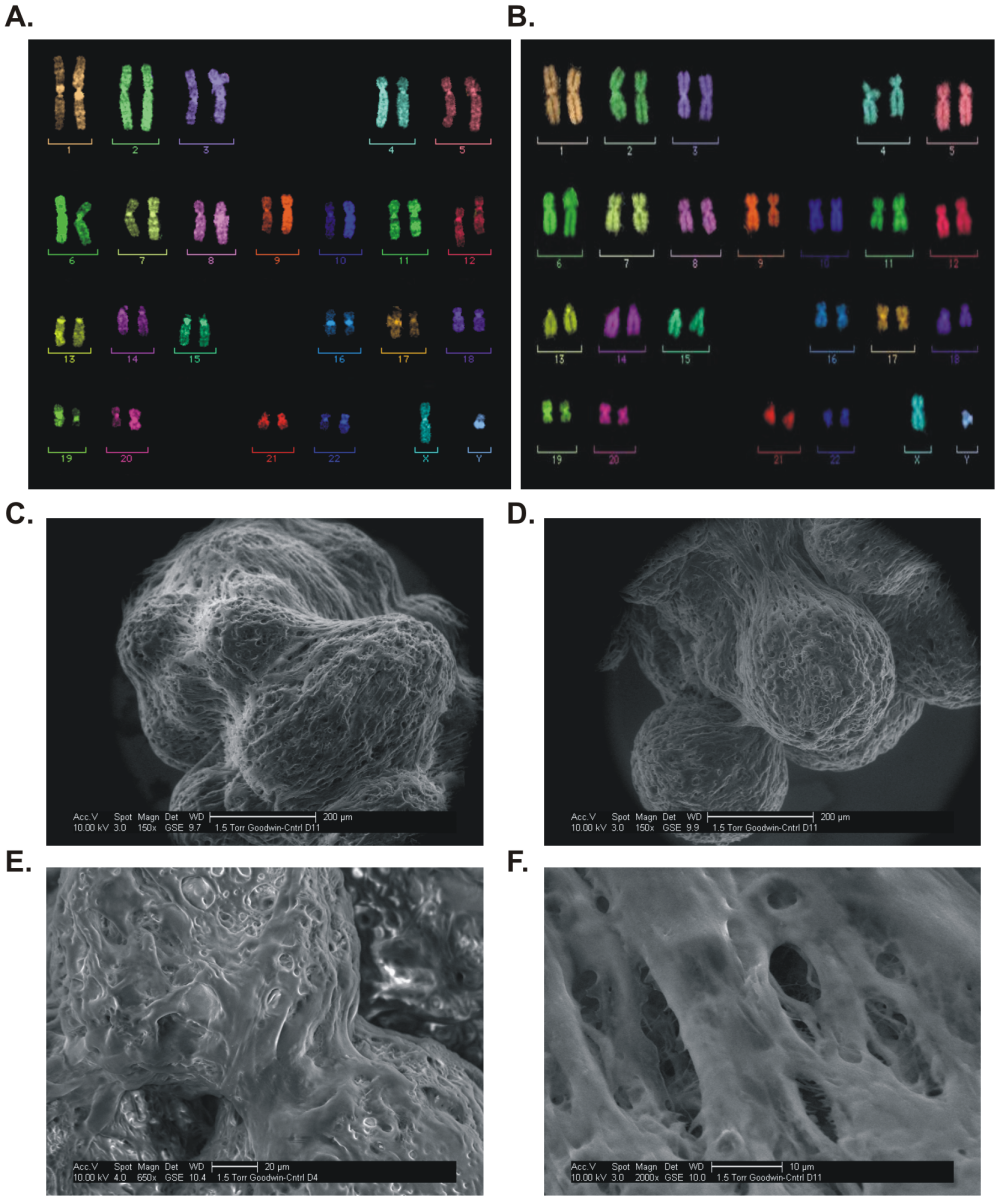
NHNP TLAs are genetically stable for at least 180 days and form tissue like assemblies. (A) mFISH analysis of passage 1 NHNP cells (left panel) and (B) NHNP TLAs after 180 days in culture (right panel). Representative metaphase chromosomes labeled with Spectra Vysion Tm probes are shown. (C–F) Environmental scanning electron micrographs with 150× (C, D), 650× (E) and 2000× (F) magnifications illustrate the relative size, density, and complexity of TLAs. Scale bars are shown in each image.

**Figure 2:**
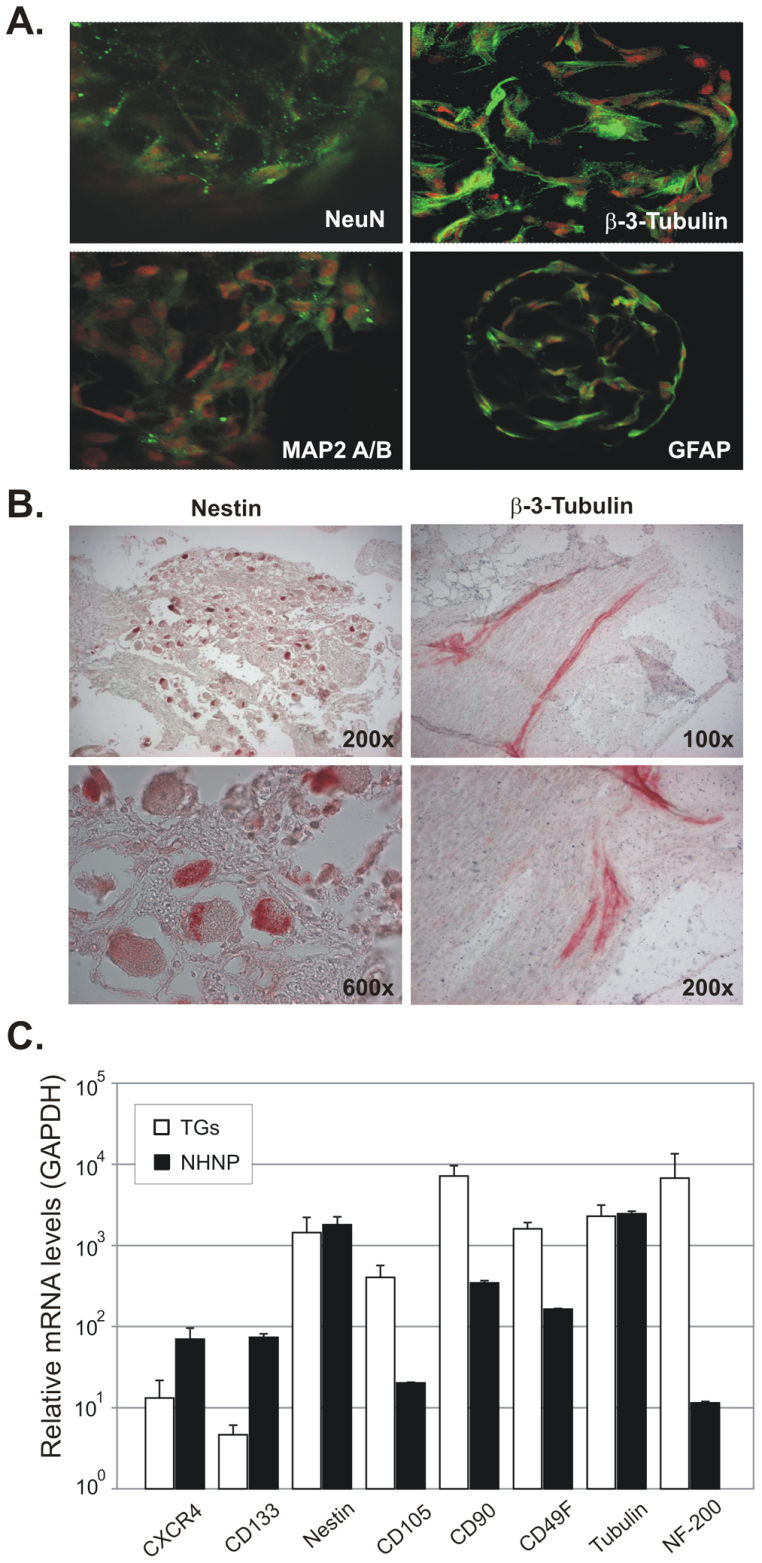
Expression of neuronal markers in NHNP TLAs and human trigeminal ganglia (TG). (A) The neuronal markers Neuron-Specific Nuclear Protein (NeuN), Microtubule Associated Protein A&B (MAP2 A/B), β-Tubulin-III and Glial Fibrillary Acidic Protein (GFAP) were detected in NHNP TLAs by confocal microscopy (green). Nuclei were visualized using Alexa Fluor 488 (red). Representative images are shown at 400× magnification. (B) Expression of Nestin and β-Tubulin-III in human TGs at indicated magnification. (C) Expression levels of CXCR4, CD133, Nestin, CD105, CD90, CD49f and β-Tubulin-III in three primary TGs (white bars) obtained from two donors or NHNP TLAs (black bars). Data is shown as mean mRNA copies relative to GAPDH with standard deviations (error bars).

NHNP cells were cultured in 3D on a scaffold of Cultispher beads in a rotating wall vessel (RWV) bioreactor and analyzed by multicolor fluorescence *in situ* hybridization (mFISH), flow cytometry, confocal microscopy and RT-PCR to assess their genetic stability as compared with 2D NHNP cell cultures. Comparison of 2D and 3D cultured NHNP cells by mFISH respectively showed no chromosomal rearrangements or breaks, indicating genetic stability of NHNP TLAs for at least a six month period in culture (Fig. 1A–B). Flow cytometry analysis confirmed that after 180 days in culture, NHNP TLAs expressed neuronal progenitor markers CXCR4, CD133, CD105-Endoglin, CD 90-Thy-1 and CD49f-α6 Integrin at levels comparable to the parental NHNP (2D) cell population (Table 1). Markers of hematopoietic differentiation, CD38^+^ (Table 1) and CD45^+^ (data not shown) were not detected in either culture. An example of the tissue-like complexity of the NHNP TLA constructs is shown by environmental scanning electron micrographs (ESEM) in Fig. 1C–F, which illustrate the relative size, density, and indistinguishable nature of the individual neural cells in the tissue assembly. In addition, confocal microscopy confirmed that NHNP TLAs expressed mature neuronal markers: neuron-specific nuclear protein (NeuN), β-Tubulin-III, microtubule associated protein A&B (MAP 2 A&B), and glial fibrillary acidic protein (GFAP) (Fig. 2A). TLA microcarrier substrates alone did not show autofluorescence (data not shown). Similarly, progenitor (Nestin) and mature (β-Tubulin-III) neuronal markers were detected by immunohistochemistry in human TG removed at autopsy (Fig. 2B). Quantitative analysis of mRNA expression revealed higher levels of the early progenitor markers CXCR4 and CD133 in NHNP TLAs than in human TG (Fig. 2C) even though only few NHNP cells expressed the two proteins (Table 1). Later stage developmental markers, CD105, CD90 and CD49f, were expressed to a lesser extent in NHNP TLAs than in human TG, suggesting that NHNP TLAs are less differentiated than primary TGs. Neuronal markers Nestin and β-Tubulin-III were expressed at comparable levels in TG and NHNP TLAs (Fig. 2C). Lower expression levels in the NHNP TLAs than in human TG were observed for the neuronal marker neurofilament 200 (NF-200), again indicating NHNP TLAs are generally less differentiated than human TG. Taken together, our data indicated that NHNP TLAs, which had undergone 18–20 fold expansion over the three-month time period, share some similarities but are less differentiated when compared to mature human TGs removed at autopsy.

**Table 1:** Marker protein expression as measured by flow cytometry demonstrates consistent expression of early progenitor and neuronal cell markers during the course of the 180 day experiment.

	% positive 2-D NHNP	% positive 3-D NHNP	Marker description
CXCR4	2.0	8.0	neural progenitor marker
CD133	4.0	5.0	primitive neural stem cell/HSC*
CD105 (Endoglin)	98.0	96.0	neural stem cell/MSC**
CD90 (Thy-1)	99.0	91.0	neural progenitor cell/HSC*/MSC**
CD49f (α6 integrin)	98.0	99.0	early progenitor maturation regulated by CD90
CD34	2.0	1.0	early hematopoietic marker (negative control)
CD38	0.0	0.0	cell activation/differentiation (negative control)

* HSC: hematopoietic stem cells.

** MSC: mesenchymal stem cells.

### Generation and characterization of fluorescently-labeled VZV

**Figure 3:**
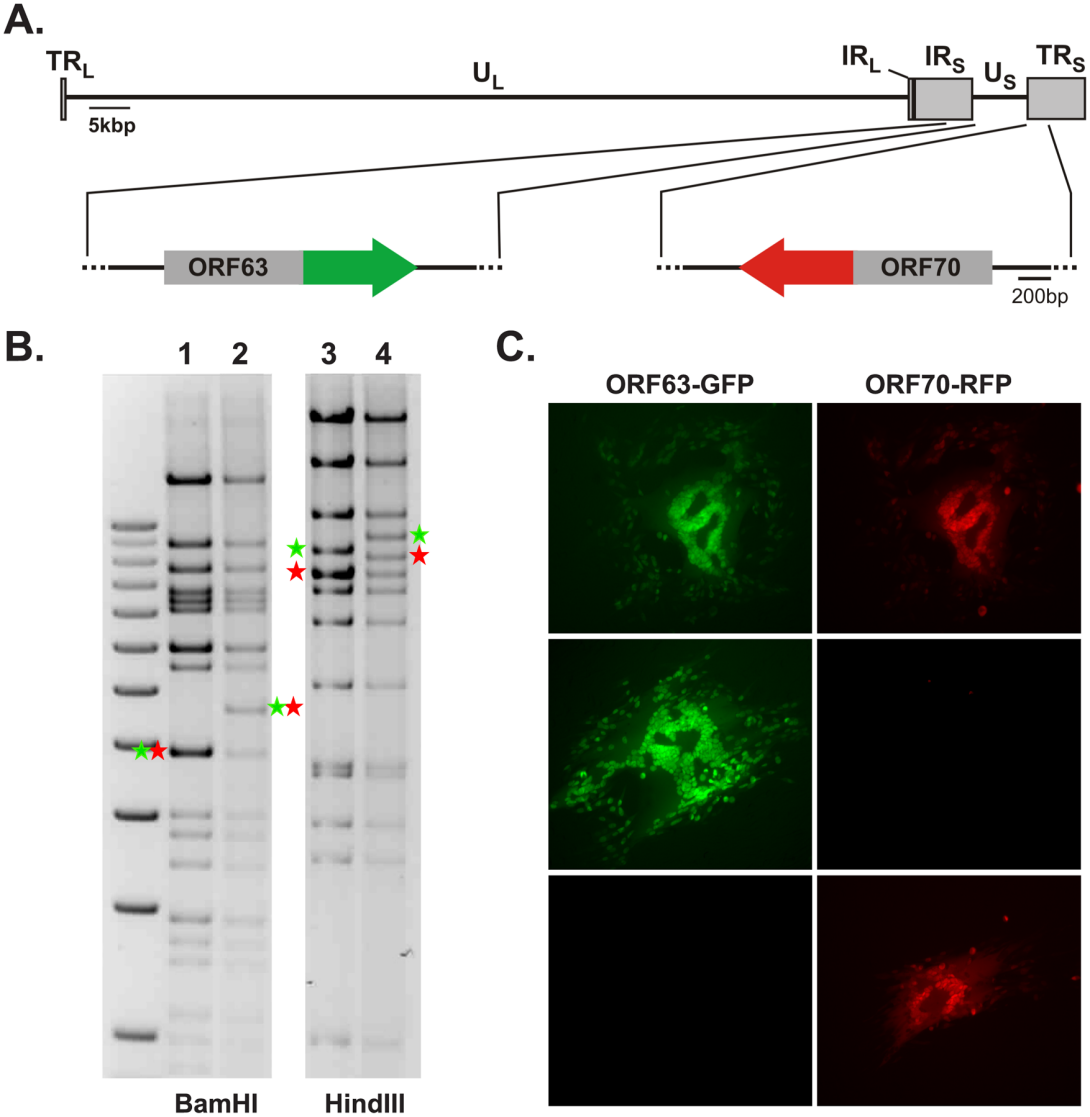
Generation of fluorescently labeled VZV P-Oka facilitates analysis of ORF63 and ORF70 expression. (A) Schematic representation of the VZV genome with a focus on the regions containing ORF63 and ORF70. Insertion of eGFP (green arrow) and mRFP (red arrow) at the C-terminus of ORF63 and ORF70 are indicated respectively. (B) Restriction fragment length polymorphism (RFLP) analysis of parental pP-Oka (lane 1 and 3) and p63G/70R (lane 2 and 4) BAC DNA digested with indicated enzymes. Green and red asterisks indicate fragments containing ORF63 and ORF70 respectively that increase in size upon insertion of eGFP and mRFP. (C) Florescence microscopy images of VZV plaques detecting ORF63-eGFP and ORF70-mRFP expression after BAC DNA transfections (upper panel) and upon passaging of the virus (middle and lower panel).

**Figure 4:**
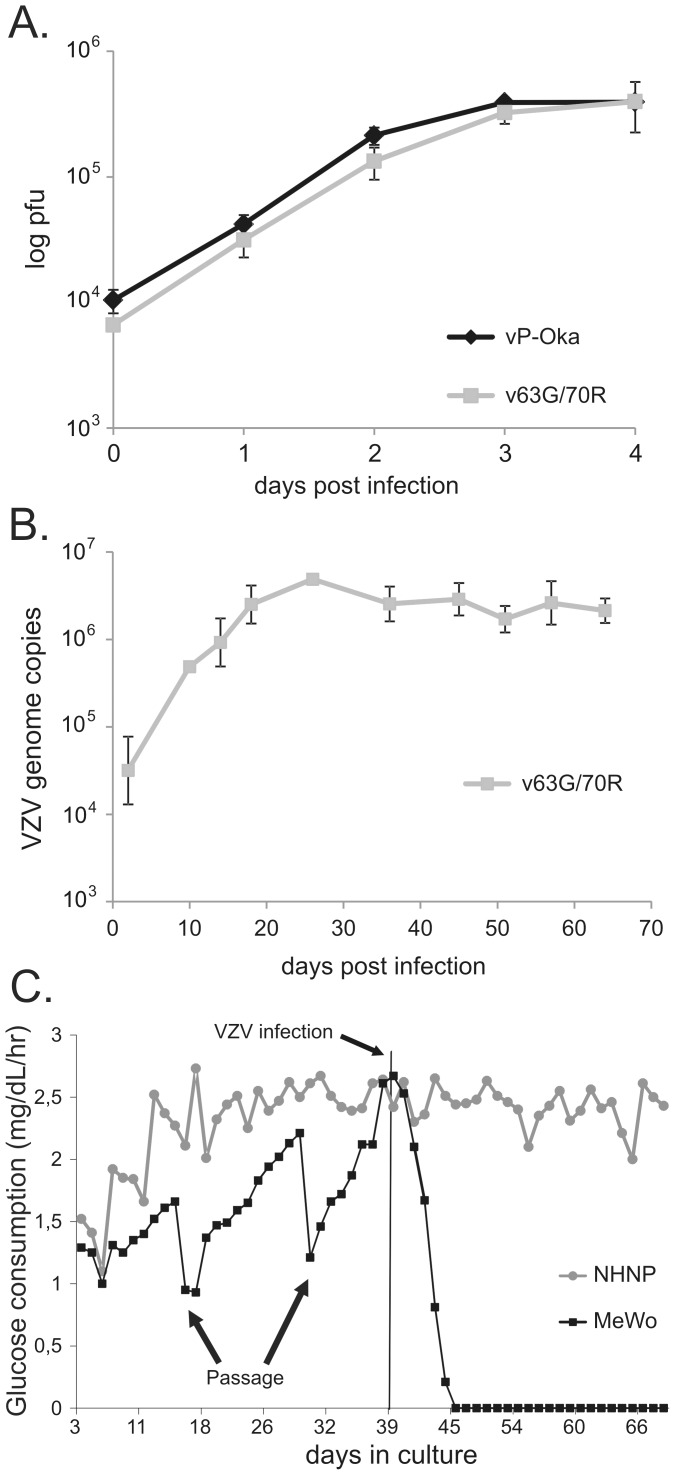
v63G/70R replicates comparable to parental virus in vitro. (A) Replication kinetics of parental P-Oka (rOKA) and v63G/70R in MeWo cells. Data is shown as mean viral titers with standard deviations (error bars). (B) Replication of v63G/70R in NHNP TLAs. NHNP TLAs were infected with v63G/70R in triplicate, samples removed at the indicated time points and analyzed by qPCR. Data is shown as mean viral genome copies with standard deviations (error bars). (C) Daily glucose utilization of MeWo cells and NHNP TLAs prior and post infection with v63G/70R at day 39. Passages of MeWo cells are indicated with an arrow. Data is shown as mean glucose utilization levels with standard deviations (error bars).

Both copies of the diploid VZV gene ORF63/70 encoding the immediate-early protein 63 (IE63) were mutated to identify expression originating from each gene. The fluorescent proteins eGFP and mRFP were inserted at the C-terminus of ORF63 and 70, respectively, in the parental VZV Oka strain. For genetic manipulations, an infectious BAC clone (pP-Oka) was used and the insertion of the marker genes resulted in recombinant BAC p63G/70R (Fig. 3A). Correct incorporation of eGFP and mRFP coding sequences was confirmed by PCR, DNA sequencing and multiple RFLP analyses (Fig. 3B). The p63G/70R BAC was then transfected into MeWo cells, resulting in reconstitution of the recombinant virus (v63G/70R). Upon virus reconstitution, ORF63-eGFP and ORF70-mRFP expression was readily detected in all plaques (Fig. 3C, upper panel), confirming that both loci were expressed in infected MeWo cells. Upon subsequent passages of v63G/70R, plaques developed in which only either eGFP or mRFP were expressed (Fig. 3C, middle and lower panel), suggesting that recombination between the IR_S_ and TR_S_ regions had occurred. PCR analysis of multiple isolated plaques positive for only eGFP or mRFP indicated homogeneity in the fluorescent tag, thus confirming that both ORF63 and ORF70 contained the same mutation (data not shown), and confirming the suspected recombination. Growth kinetics showed no significant effect on virus replication due to insertion of the fluorescent proteins into ORF63/70 (Fig. 4A). Taken together, our data indicated that recombination between the IR_S_ and TR_S_ region occurred frequently in cultured MeWo cells, which resulted in a stable replacement of IR_S_ or TR_S_ with their respective counterparts, TR_S_ or IR_S_, respectively.

### v63G/70R infection of NHNP TLAs

**Figure 5:**
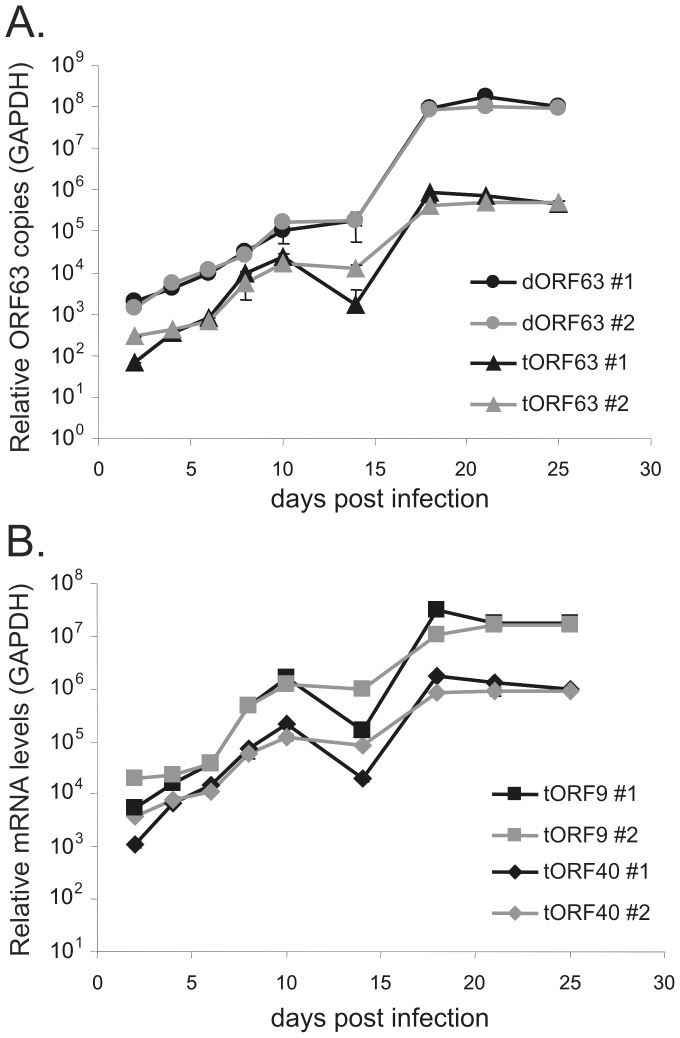
VZV gene transcription in infected NHNP TLAs. Two independent NHNP TLAs were established, infected with v63G/70R and sampled at indicated times post infection. A) qPCR analysis of VZV DNA genome copies (dORF63) and qRT-PCR of ORF63/70 transcript copies (tORF63) normalized against corresponding GAPDH copies. (B) qRT-PCR analysis of VZV ORF40 (tORF40) and ORF9 (tORF9) transcript copies normalized against GAPDH mRNA copies. Data of two independent experiments analyzed in triplicate is shown as mean relative copies and was analyzed using ΔΔCt analyses.

Cell-free v63G/70R efficiently infected NHNP TLAs, as evidenced by an approximate 50-fold increase in VZV genome copies from 0 to 18 days post-infection (dpi). After 18 dpi, VZV genome copy numbers remained constant (Fig. 4B). To compare the overall health of VZV infected MeWo cells grown in 2D to VZV infected NHNP TLAs grown in 3D, glucose utilization pre- and post-VZV infection was monitored (Fig. 4C). Each culture was maintained initially for 39 days to establish a baseline glucose consumption rate. Upon v63G/70R infection, glucose utilization rapidly declined in MeWo cells, most likely a consequence of lytic VZV replication as seen microscopically by extensive VZV-induced cytopathic effects (data not shown). In contrast, glucose uptake in NHNP TLAs was not altered as a response to VZV infection (Fig. 4C), suggesting that limited, if any, lytic VZV infection occurred. Quantitative RT-PCR was performed to determine if the virus genome was transcribed in infected NHNP TLAs. Transcript levels of the immediate-early gene ORF63 increased over time and correlated to the amount of VZV genome copies (Fig. 5A). Further analysis showed that late VZV gene ORF 9 and ORF 40 transcripts also increased in abundance (Fig. 5B).

To determine if infectious virus was released from infected NHNP TLAs, cell-free supernatants from VZV infected NHNP TLAs were titrated using permissive MeWo cells and plaques stained using an anti-VZV IE63 antibody. Shortly after inoculation of the NHNP TLA culture with VZV, high levels of cell-free virus were detected (Table 2). Each day post infection, 80% of the culture medium was replaced, resulting in a rapid removal of cell-free virus from the reactor. However, low-level release of cell-free VZV was detected on 6, 10, 25 and 35 dpi, while no infectious virus was detected on 14, 21 and 30 dpi, indicating intermittent *de novo* production and release of infectious virus (Table 2). It is noteworthy that the NHNP TLAs remained intact and healthy despite apparent virus production.

**Table 2:** Release of infectious virus into VZV infected NHNP TLA tissue culture fluid.

days post infection	plaque forming units per ml (5 wells)				
	1	2	3	4	5
0	371	351	207	402	457
2	5	2	2	1	2
4	3	0	0	2	2
6	1	2	2	2	1
10	0	1	0	0	2
14	0	0	0	0	0
21	0	0	0	0	0
25	2	1	1	3	2
30	0	0	0	0	0
35	0	0	1	1	0

**Figure 6:**
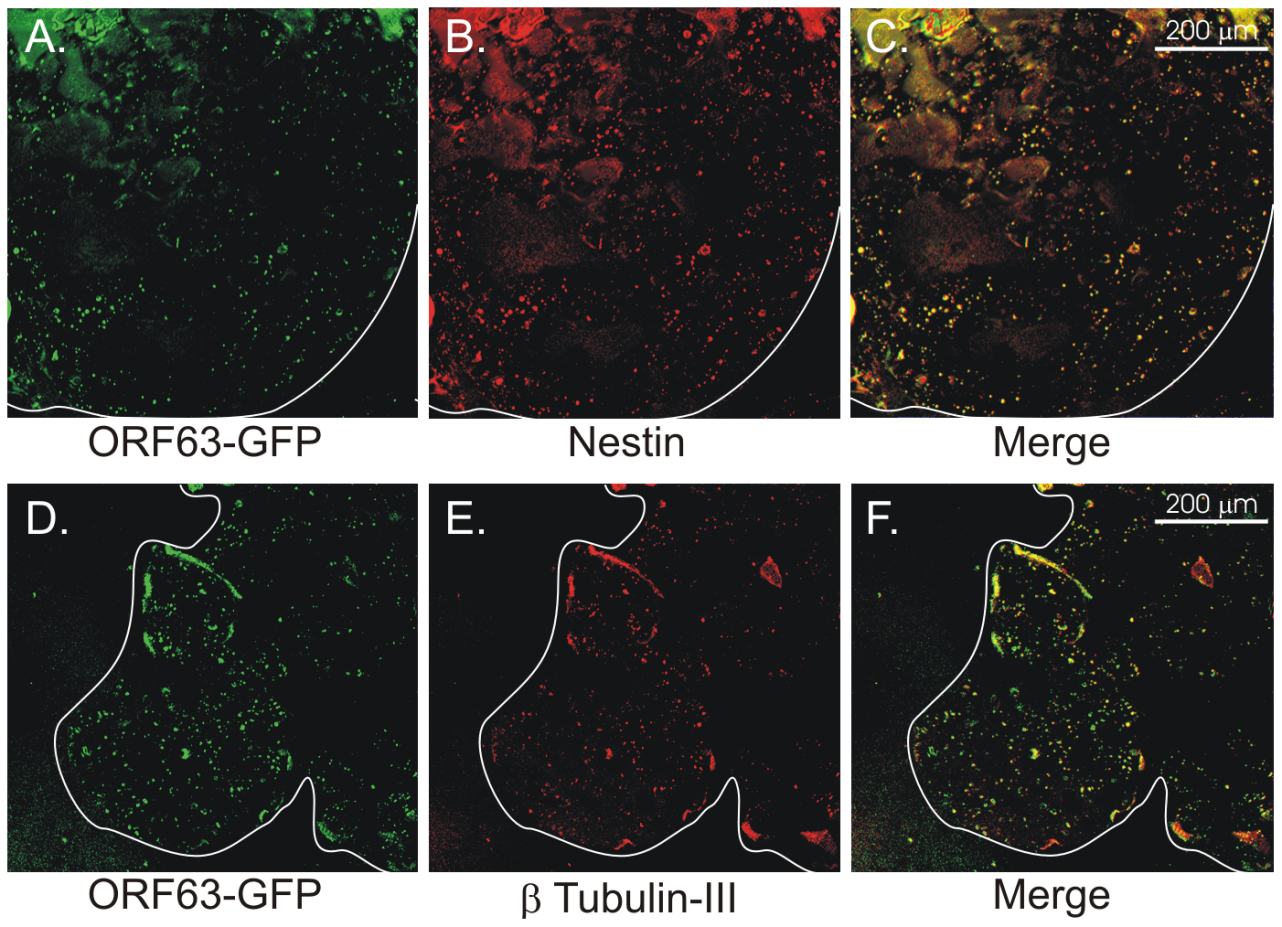
Infected NHNP TLA cells express progenitor and mature neuronal markers. NHNP TLAs were infected with v63G/70R, fixed 21 days post-infection and stained for the progenitor marker Nestin (upper panels, A–C) and the mature marker β-Tubulin-III (lower panels, D–F). Representative confocal images are shown at 40× magnification. The outline of the TLAs (white line) and scale bars are indicated.

Confocal analysis of NHNP TLAs infected with VZV containing eGFP fused to both ORF63 and ORF70 at 27 dpi revealed that the progenitor neuronal marker Nestin (Fig. 6 A–C) and the mature neuronal marker β-Tubulin-III colocalized with ORF63-eGFP (Fig. 6D–F). Taken together, our data demonstrates that VZV infection of NHNP TLAs results in prolonged accumulation of virus DNA, mRNA and sporadic release of very small amounts of infectious cell-free virus.

### Stable maintenance of the VZV genome in NHNP TLAs

**Figure 7:**
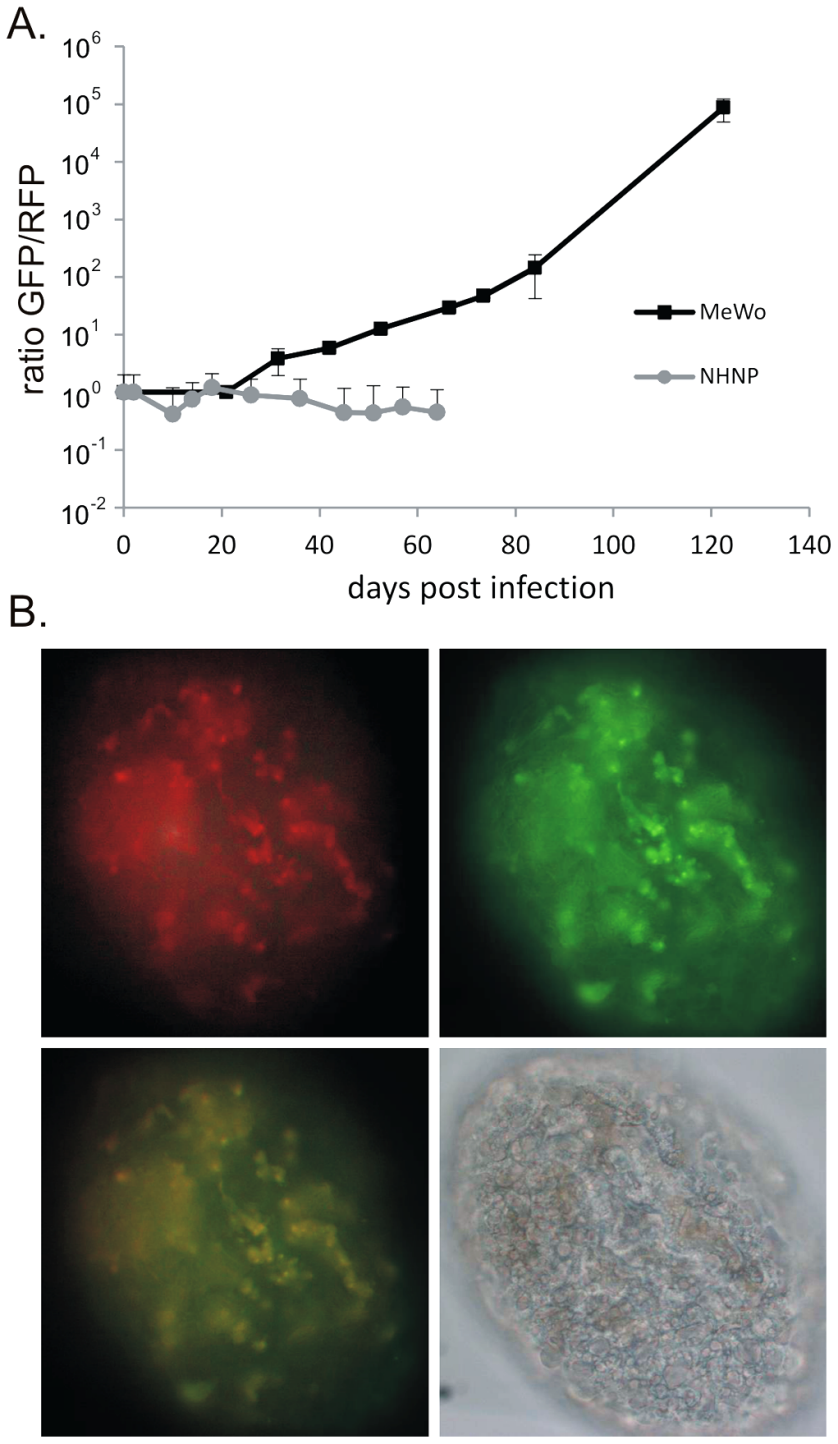
The VZV genome is genetically stable in infected NHNP TLAs. (A) MeWo cells and NHNP TLAs were infected with v63G/70R and propagated for the indicated time frames. Infected MeWo cells were passaged every 3–4 days, while infected NHNP TLAs were maintained by the replenishment of medium as required. At indicated time points, samples from the infected MeWo cells and NHNP TLAs were removed, total DNA extracted and DNA copies of ORF63-eGFP and ORF70-mRFP determined by qPCR. Data are shown as mean ratio of ORF63-eGFP to ORF70-mRFP determined in triplicate from two independent experiments in MeWo cells and 4 independent NHNP TLAs. (B) ORF63-eGFP and ORF70-mRFP expression (upper row) in a representative NHNP TLA at 27 days post infection with v63G/70R. An overlay of eGFP and mRFP fluorescence as well as a bright field image (lower row) of the NHNP TLA is shown at 40× magnification.

Recombination between inverted repeat regions in the genome of alphaherpesviruses during lytic replication is well documented [20], [21]. To determine if VZV DNA recombination occurred in MeWo (2D) or NHNP TLAs (3D) cultures, we determined the recombination frequency between ORF63 and ORF70 by qPCR. During lytic replication in MeWo cells, recombination within the VZV genome that resulted in a replacement of ORF70-mRFP with ORF63-eGFP occurred frequently (Fig. 7A), indicating a selective pressure against the ORF70-mRFP fusion protein. In contrast, the GFP/RFP ratios in v63G/70R infected NHNP TLAs remained unaltered for at least 69 days in culture, suggesting that the VZV genome is stably maintained in NHNP TLAs. In addition, confocal microscopy confirmed that both ORF63-eGFP and ORF70-mRFP are expressed in NHNP TLA cultures infected with v63G/70R (Fig. 7B). Our data indicates a stable viral genome is preserved for an extended period in NHNP TLAs.

## Discussion

Establishment of latent infection and subsequent reactivation is integral to the alphaherpesvirus life cycle and ensures that virus is maintained in the population by intermittent virus production and transmission [22]. However, VZV appears to be unique among alphaherpesviruses with respect to establishment of and reactivation from the persistent/latent state [23]–[26], emphasizing the need for accurate nomenclature. VZV-host interactions are classified as acute, “rapid production of infectious virions followed by rapid resolution and elimination of infection”, or persistent, “virus particles or products continue to be produced for long periods in which virions are continuously or intermittently produced” [27]. In most human cells in culture, VZV infection is acute and cells succumb to virus infection within 3–5 days most likely through apoptosis [28]–[31]. The low ratio (1∶40,000) of infectious to defective VZV particles indicates that production of complete virions is extremely inefficient [32]. Human neurons are known to be latently infected by VZV, but the lack of a suitable animal model has hindered investigations into this exceptional relationship between VZV and a host cell or organ. Our common understanding is that VZV infection of neurons results in latency. Latency is an extreme variant of persistent infection where, as exemplified by herpes simplex virus type 1 (HSV-1), “infectious virions can no longer be isolated” [27]. During latency, most HSV-1 genes are silenced through epigenetic modification of resident histones or by virus-specific miRNAs [33]–[35]. Therefore, HSV-1 gene transcription in infected neurons is restricted to the latency-associated transcript (LAT) at the cellular level, while no viral proteins and infectious virions are produced [36]–[38].

According to the above definitions and following the example provided by HSV-1, the prototype alphaherpesvirus, VZV infection of NHNP TLAs maintained in 3D cultures is persistent and not latent. While VZV DNA is present and virus-induced CPE was not detected either microscopically or by monitoring total glucose uptake, VZV genes are transcribed, IE63 is translated, and small amounts of infectious virions are intermittently produced. The observation that the quantity of late VZV transcripts directly correlates to IE63 transcription and viral genome copies, suggests that many cells could express these transcripts and potentially produce infectious virus. Alternatively, late transcripts as well as infectious cell-free virions could be derived from very few cells that productively replicate VZV. While at least 12 VZV genes are transcribed in human ganglia removed at autopsy [39]–[41] and IE63 protein production is commonly detected [42], it is known that the extent of VZV transcription increases with longer times between death and tissue analysis [43]. Hence, it has been argued that transcription of multiple virus genes detected in human ganglia most likely reflects reactivation of latent virus genomes [44], [45]. Thus, while multiple viral genes are transcribed in infected NHNP TLAs and in human trigeminal ganglia containing VZV DNA at autopsy, the former is a long-term permissive infection and the later most likely reflects early stage virus reactivation.

Our current work highlights the importance of NHNP TLAs in 3D culture for the study of VZV persistent infection, latency and reactivation. The ability to maintain VZV infection for extended times along with the ease of experimental manipulation allows us to investigate the VZV life cycle in an *in vitro* system for the first time. Our 3-month experiment was operator-terminated, and the healthy cultures still contained VZV DNA. As such, infected NHNP TLAs in 3D culture provide an excellent means to investigate chronic virus-neuron interactions. An additional attribute of the 3D cultures is that they allow introduction of autologous VZV-specific CD8 T-cells with the aim of immune surveillance that would remove cells actively producing virus. Previous studies on HSV-1 showed that CD8 T-cell surveillance within latently infected human and mouse trigeminal ganglia may help maintain latent infection [46]–[56]. This in turn could be also true for VZV and could allow the establishment of latently infected NHNP cultures by the addition of CD8 T-cells.

The NHNP TLAs system developed here is based on growth on inert support microspheres that proliferate and remain viable in a 3D fluid environment using an optimized GTSF-2 medium. Various TLAs systems have been shown to exhibit morphologies, growth characteristics, and cytokine expression similar to human lung, liver, and small intestine [57]–[62]. Therefore, TLAs provide a promising platform to model viral infections of human cells in a 3D environment that is similar to the natural tissue. We have shown previously that human bronchio-tracheal tissue maintained as 3D-TLAs (HLEMs) can be used to investigate respiratory syncytial virus (RSV), parainfluenza virus (PIV3) and severe acute respiratory syndrome (SARS) [57], [60], [63], [64] infections. HLEMs can be maintained for at least two months and can facilitate the discrimination between pathogenic and attenuated RSV and PIV3 strains based on viral replication efficiencies and inflammatory responses. In addition, SARS was shown to replicate for up to two weeks in HLEMs without TLA destruction [57], [63], [64]. The NHNP TLAs we constructed can also be used to investigate other neurotropic viruses (West Nile, Dengue and Vesicular Stomatitis Virus [VSV]) [65]–[66, unpublished data].

As mentioned previously, VZV infection of most human cells results in acute cell death. This also occurs in neuronal cultures maintained in 2D, unless they are devoid of non-neuronal cells. Our 3D neuronal TLA cultures are unique in that VZV infection did not destroy the tissue-like assemblies even though low amounts of infectious virus were sporadically released and even when a mixed neural progenitor cell population was present. In this respect, neuronal progenitor cell cultures maintained in 3D are fundamentally different from the same cells maintained in 2D [17], [19]. Therefore, this newly established NHNP TLA 3D system provides the unique opportunity to investigate neurotropic viruses in a neuronal setting over extended periods of time.

## Materials and Methods

### NHNP TLA 3D cell culture system

NHNP cells were obtained from Lonza (Walkersville, MD, USA) and propagated in GTSF-2, a unique media containing glucose, galactose and fructose supplemented with 10% fetal bovine serum (FBS), at 37°C under a 5% CO_2_ atmosphere [67]–[69]. NHNP cells were initially grown as monolayers in human fibronectin-coated flasks (BD Biosciences, San Jose, CA) and pooled from at least five donors, as described previously [70]. NHNP cell cultures were expanded, tested for viral contaminants as pre-certified by the manufacturer's production criteria (Lonza), and cryopreserved in liquid nitrogen. Three-dimensional (3D) NHNP TLAs were generated by seeding 3×10^5^ NHNP cells/ml onto 3 mg/ml Cultispher beads (Sigma-Aldrich, St. Louis, MO) into a 55 ml rotating wall vessel bioreactor (RWV; Synthecon, Houston, TX) and grown at 37°C under a 5% CO_2_ atmosphere. Cells were allowed to attach to the beads for 48 h in the bioreactor before re-feeding with GTSF-2 containing 10% FBS. To maintain the TLA cultures within normal human physiological blood chemistry parameters (pH 7.2 and a glucose concentration of 80–120 mg/dL), 20–90% of the media was replaced as required with fresh GTSF-2 media every 48 h, facilitating efficient TLA tissue growth and maturation prior to VZV infection. All metabolic determinations were made using an iStat hand held blood gas analyzer (Abbott Laboratories, Abbott Park, IL).

### Propagation of MeWo cells and VZV

Human melanoma cells (MeWo, American Type Culture Collection, ATCC, Manassas, VA.) were propagated in Dulbecco's minimal essential medium supplemented with 10% fetal bovine serum, 100 U/ml penicillin and 0.1 mg/ml streptomycin (Sigma-Aldrich, St. Louis, MO) at 37°C under 5% CO_2_ [71]. Wild-type and recombinant viruses were passaged on MeWo cells by co-cultivation of infected with uninfected cells at a ratio of 1/5 [72]. MeWo cells for the infection of NHNP cultures were adapted to GTSF-2 medium over two passages prior to harvest of VZV.

### Preparation of cell-free VZV

Cell-free VZV [73] was used for the NHNP TLA infections to avoid transfer of infected MeWo cells to the TLA culture. Briefly, infected cells were harvested at 96 h post-infection (p.i.) and resuspended in reticulocyte standard buffer (10 mM NaCl, 1.5 mM MgCl_2_, 10 mM Tris-HCl, pH 7.4). Cells were disrupted by Dounce (type A) homogenization (Cole-Parmer, Vernon Hills, IL), debris removed by centrifugation at 900×*g* for 15 min and virus containing supernatant filtered using a 1.0 µm Millex filter unit (Millipore Billerica, MA.).

### Infection of 3D NHNP TLA cultures

NHNP TLAs were infected in the RWV with cell-free VZV at a multiplicity of infection (MOI) of 0.1 by absorption at room temperature for 30 min in 20 ml GTSF-2. Then, the RWVs were filled with fresh GTSF-2/10% FBS and transferred to a humidified incubator with a 5% CO_2_ atmosphere at 37°C. Every 24 h.p.i., ∼80% of the culture media was replaced with fresh GTSF-2 containing 10% FBS. Samples were collected approximately every other day for ∼70 days to determine viral genome copies as described below. Supernatant of infected NHNP TLAs was removed, centrifuged to remove cells and debris and titrated in fresh MeWo cells to determine the amount of infectious VZV virions in the NHNP culture media.

### Metaphase fluorescence in situ hybridization (mFISH) analysis

Multicolor mFISH for the analysis of chromosome integrity was conducted as described [74], [75]. Briefly, NHNP cells and TLAs were cultured as previously stated, with the exception of being subcultured at a low density for 36 h. Cultures were incubated for 20 min with 50 nM calyculin-A (Waco Chemicals, Japan), treated with 0.075 M KCl hypotonic solution at 37°C for 20 min, fixed in methanol/acetic acid (3∶1 vol/vol) and stored at −20°C. Chromosome spreads were prepared as described [76], [77]. Chromosome-containing slides were hybridized with Spectra Vysion Tm probes (Vysis, Downers Grove, IL) to detect specific chromosome pairs. Chromosomes were stained with DAPI and analyzed using a Zeiss Axioplan fluorescence microscope.

### Environmental scanning electron microscopy

Sample preparation, scanning and analysis of the TLAs was performed as described previously [78], [79]. Images of NHNP TLAs were taken using a Philips XL 30 ESEM (FEI Co., Hillsboro, OR), at 150×, 650× and 2000× magnifications to illustrate the complexity and the tissue–like nature of the cultures.

### Confocal and flow cytometry analyses

Neuronal markers were detected by indirect immunofluorescence using anti-human CXCR4, CD133, Nestin, CD105 (Endoglin), CD90 (Thy-1) CD49f (ITGa6) and β-Tubulin-III at the following dilutions: CXCR4 50 µg/ml, CD133 25 µg/ml, Nestin 50 µg/ml, CD105 Endoglin 50 µg/ml, CD90 Thy-1 50 µg/ml, CD49f (ITGa6) 25 µg/ml, β-Tubulin-III 25 µg/ml, CD34^+^ 25 µg/ml, and CD38 25 µg/ml. All reagents were obtained from R&D Systems (Minneapolis, MN.) with the exception of CXCR4 obtained through ABCAM (Cambridge, MA.). Confocal and flow cytometry used the same reagents and confocal analyses were performed as described previously [57], [60], [80]. Flow cytometry followed the method of Morrison et al. [81] with minor modifications. Briefly, NHNP TLAs from RWV culture vessels were collected into a 50 mL conical tube and dissociated in 15 mL of 2% trypsin for <5 min, just long enough to dislodge cells. Trypsin was inactivated with 35 mL cold 10% FBS in PBS. Culture beads were allowed to sediment and free floating cells were transferred into a fresh conical tube, washed twice in PBS, centrifuged for 3 min at 600×*g*. The soft cell pellet was resuspended in GTSF-2 and permitted to recover for 1 h at 37°C. After recovery, viable cells (trypan blue exclusion) were counted and aliquots (∼1×10^5^) were washed 2× in PBS and used for flow cytometry. Fluorochromes were added and incubated 45 min at 4°C in PBS, washed 2× in PBS at 4°C and brought to 400 µL with PBS. All analysis was done on freshly conjugated cells. Samples were either analyzed by confocal microscopy on a Leica TCS/SP2 3-laser confocal microscope or a Beckman Coulter XL2 Flow Cytometer (Table 1).

### Human trigeminal ganglia and immunohistochemistry

Collection and use of human cadaver tissue was approved by the University of Colorado Internal Review Board and removed only after obtaining informed consent from next-of-kin (Multi Institution Review Board Document No. B182). Harvest of cadaver tissues is not considered human research as determined by the IRB. Trigeminal ganglia were fixed in 4% paraformaldehyde overnight, dehydrated through graded ethanol, and embedded in paraffin. Five micrometer sections were collected on Superfrost (Cole-Parmer, Vernon Hills, IL) slides and fixed at 72°C for 30 min. Samples were deparaffinized in xylene and rehydrated in graded ethanol. Antigen was retrieved by soaking the sections in 10 mM sodium citrate (pH 6.0) in a steamer for 25 min. Slides were blocked in 5% normal goat serum for 1 h at room temperature and then incubated with mouse anti-Nestin monoclonal (1∶1,000 dilution; BD Transduction Labs, San Jose, CA) or mouse anti-β-Tubulin-III monoclonal (1∶200 dilution; Cell Signaling, Danvers, MA) antibody overnight at 4°C. Slides were washed in PBS and biotin-conjugated goat anti-mouse IgG (1∶1,000 dilution; Dako, Carpinteria, CA) was applied for 1 h at room temperature. After secondary antibody application, slides were PBS washed and incubated with diluted alkaline phosphatase-conjugated streptavidin (BD Biosciences, San Diego, CA) for 1 h. The color reaction was developed for 2 min using the fresh fuchsin substrate system (Dako, Carpinteria, CA) in the presence of levamisole at a final concentration of 24 µg/ml. All images were acquired using Axiovision (Zeiss) digital imaging software with a Nikon Eclipse E800 microscope.

### Nucleic acid quantification

DNA and RNA were extracted from cell pellets using DNeasy and RNeasy kits according to manufacturer's protocol (Qiagen, Valencia, CA). Complementary DNA was synthesized from 100 ng of DNase-treated RNA using the Transcriptor System (Roche Diagnostics, Mannheim, Germany) as previously described [82]. Specific primers and probes for TaqMan and SYBR PCR were obtained from IDT (Cedar Rapids, IW) to quantify VZV ORF9, ORF 40, ORF 63/70 and the cellular GAPDH gene. To differentiate between VZV DNA and transcripts containing eGFP or mRFP fused to the 3′-end of ORF63/70, we used specific primers and probes for each target (Table 3). PCR amplification using the 7500-Fast real-time PCR system (Applied Biosystems, Foster City, CA) was previously described [83]. All primers and probes are shown in Table 3.

**Table 3:** Primers used for qPCR and the construction of virus mutants.

construct		sequence (5′→3′)
63/70EGFP	for	GTCGACACGAAGCCCCGCGCCGGCATGATATACCGCCCCCCCATGGCGTGGTGAGCAAGGGCGAGGAGCT
	rev	ACATCAAAAAAAGACACGAGCCAAACCATTGTATTTATTTATAAAGACTACTTGTACAGCTCGTCCATGCCG
63/70mRFP	for	GTCGACACGAAGCCCCGCGCCGGCATGATATACCGCCCCCCCATGGCGTGGCCTCCTCCGAGGACGTCATCAAG
	rev	ACATCAAAAAAAGACACGAGCCAAACCATTGTATTTATTTATAAAGACTACAAGGCGCCGGTGGAGTGGC
ORF63 seq	for	CCTGGTTACCCAGGCCGTGC
	rev	GAGCGATACGCGGGTGCAG
ORF70 seq	for	CAAGTCCCCGTATAACCAAAGCATG
	rev	GAGCGATACGCGGGTGCAG
**qPCR target**		
CXCR4	for	GGCCGACCTCCTCTTTGTC
	rev	TTGCCACGGCATCAACTG
CD105 (Endoglin)	for	CCGCGCTTCAGCTTCCT
	rev	GAGGGTGCCGGTTTTGG
CD49f (ITGa6)	for	GATCCCGGCCTGTGATTAATATT
	rev	CTGGCGGAGGTCAATTCTGT
Nestin	for	AGCCCTGACCACTCCAGTTTAG
	rev	CCCTCTATGGCTGTTTCTTTCTCT
CD133 (PROM1)	for	ATCTGCAGTGGATCGAGTTCTCT
	rev	GCGGTGGCCACAGGTTT
CD90 (Thy-1)	for	CCGCTCCCGAACCAACT
	rev	GGCGGATAAGTAGAGGACCTTCA
β-Tubulin-III	for	AACACGGATGAGACCTACTGCAT
	rev	GGGTGCGGAAGCAGATGT
GAPDH	for	CACATGGCCTCCAAGGAGTAA
	rev	TGAGGGTCTCTGTCTTCCTCT
	probe	CTGGACCACCAGCCCCAGCAAG
ORF9	for	GGGAGCAGGCGCAATTG
	rev	TTTGGTGCAGTGCTGAAGGA
	probe	CAATTGCCAGCGGGAGACC
ORF40	for	ACTTGGTAACCGCCCTTGTG
	rev	CGGGCTACATCATCCATTCC
	probe	ATGGGAAAGGCCGTCCGCGG
ORF63 (qPCR)	for	GCGCCGGCATGATATACC
	rev	GACACGAGCCAAACCATTGTA
	probe	CCCCCCATGGCGTGTAGTC
ORF63-EGFP	for	TCCGCCCTGAGCAAAGAC
	rev	GAACTCCAGCAGGACCATGTG
	probe	CAACGAGAAGCGCGAG
ORF63-RFP	for	AGACCACCTACATGGCCAAGA
	rev	TGGGAGGTGATGTCCAGCTT
	probe	CGCCTACAAGACCGACA

### Fluorescence tagging of VZV ORF63/70

VZV ORFs 63 and 70 were C-terminally labeled with the enhanced green fluorescent protein (eGFP) or the monomeric red fluorescent protein (mRFP) (Fig. 5A) using two-step Red-mediated *en passant* mutagenesis as described [84]. Briefly, the eGFP-I-*Sce*I-*aphAI* and mRFP-I-*Sce*I-*aphAI* cassette was amplified from pEP-eGFP-in and pEP-mRFP-in, respectively [84], [85] with specific primers (Table 3). PCR products were introduced into pP-Oka, an infectious BAC clone of the P-Oka strain of VZV [86], [87] by Red recombination performed in GS1783 *E.coli* cells (a kind gift from Gregory A. Smith, Northwestern University, Chicago, IL). All clones were confirmed by DNA sequencing using primer sets specific for either ORF63 or ORF70, as well as multiple restriction fragment length polymorphism analyses (RFLP), to ensure the integrity of the genome (Fig. 5B).

### Reconstitution of VZV from BAC DNA

Recombinant BAC DNA used for transfection was isolated using the plasmid Midi-prep kit according to the manufacturer's instruction (Qiagen, Valencia, CA). MeWo cells were transfected with the Lipofectamine 2000 reagent (Invitrogen, Carlsbad, CA) as described [88]. Briefly, MeWo cells were transfected with 1 µg BAC DNA and 200 ng pCMV62, a plasmid that contains the VZV IE gene ORF62 under the control of the human cytomegalovirus immediate-early promoter (kindly provided by Dr. Paul Kinchington, University of Pittsburgh Medical School). Reconstituted viruses were propagated on MeWo cells as described above and analyzed for expression of the ORF63GFP/70RFP fusion proteins using a Zeiss Axiovert 25 fluorescence microscope system. In addition, VZV containing fluorescent tags at the C-terminus of both ORF63 and ORF70 upon recombination in MeWo cells was plaque purified and some viruses contained only either the eGFP or mRFP tag after plaque purification as shown by PCR and DNA sequencing analysis.

### Growth kinetics of VZV

MeWo cells (1×10^6^) were inoculated with 100 plaque-forming units (pfu) of cell-associated virus. Infected cells were trypsinized at 24, 48, 72 or 96 h post infection (h.p.i.), titrated in 10-fold dilutions, and added to MeWo cell monolayers seeded 24 h before. The number of plaques was determined by indirect immunofluorescence using an anti-VZV antibody exactly as described earlier [89].

### 3D TLA growth kinetics and glucose consumption

Metabolic parameters of infected and uninfected NHNP TLAs were measured every 24 h over the course of the experiments to monitor a cellular development profile and to monitor the metabolic status of the tissues. Glucose consumption was determined using the iStat clinical blood gas analyzer using an EC8^+^ cartridge (Abbott Laboratories, Abbott Park, IL) according to the manufacturer's instructions [60].
